# Collapsing glomerulopathy associated with hemophagocytic syndrome in a patient with NK/T cell lymphoma 

**DOI:** 10.5414/CNCS108586

**Published:** 2016-06-27

**Authors:** Wihib Gebregeorgis, Inder Patel, Manish Thakur, Divaya Bhutani, Indryas Woldie

**Affiliations:** 1 Division of Nephrology, Department of Internal Medicine, Wayne State University, and; 2 Department of Oncology, Wayne State University, Karmanos Cancer Center, Detroit, MI, USA

**Keywords:** hemophagocytic syndrome, collapsing glomerulopathy, NK/T cell lymphoma

## Abstract

Hemophagocytic syndrome (HPS) is a rare condition caused by dysregulated activation of the immune system leading to infiltration of bone marrow and organs by nonmalignant macrophages that phagocytose blood cells. Primary HPS is caused by inherited immune dysregulation whereas secondary HPS is triggered by neoplastic, infectious or autoimmune diseases. Clinically, the syndrome presents with continuous high-grade fever in association with multi-organ involvement. Few data are available regarding renal manifestations of HPS. We report a 60-year-old patient with NK/T cell nasopharyngeal extranodal lymphoma who presented with acute kidney injury and nephrotic range proteinuria in association with fever and pancytopenia. A kidney biopsy was consistent with collapsing glomerulopathy. A final diagnosis of HPS was made on the basis of clinical, laboratory, and bone marrow biopsy findings in accordance with established diagnostic criteria. Steroid therapy was initiated. However, the patient failed to recover his renal function and remained hemodialysis-dependent. Key diagnostic and therapeutic challenges and strategies used to overcome those challenges are discussed.

## Introduction 

Hemophagocytic syndrome (HPS) is a syndrome of excessive immune activation characterized by bone marrow and organ infiltration by activated, nonmalignant macrophages, which phagocytose blood cells [1]. The cellular proliferation is accompanied by the release of pro-inflammatory cytokines [2, 3]. The excessive inflammation is thought to be caused by a lack of normal down-regulation of activated macrophages and lymphocytes [4]. HPS may be primary as observed in genetic diseases affecting the immune system or secondary to a malignancy, an autoimmune disease or a severe infection [5]. 

Clinically, common presenting features include fever, hepatosplenomegaly, lymphadenopathy, and jaundice. Other manifestations include skin rash, lung infiltration, and hemorrhagic complications. Pancytopenia, liver test abnormalities, coagulopathy with low fibrinogen levels, marked hypertriglyceridemia, and elevated serum ferritin are some of the frequent laboratory findings [6]. 

The diagnosis is confirmed by the presence of pathognomic genetic mutation or fulfillment of the diagnostic criteria established by the Histiocyte Society (Table 1) one of which is diffuse infiltration by well-differentiated macrophages, actively phagocytosing hematopoietic elements in the bone marrow, lymph nodes, liver, or spleen [7, 8]. 

Data related to renal complications are limited. Renal involvement has previously been reported in 24 adult cases, mostly as acute renal failure [9]. Nephrotic syndrome has also been described. Collapsing glomerulopathy is extremely rare with only six previous cases reported in the literature [1, 9, 10]. 

We report an unusual case of collapsing glomerulopathy associated with HPS that complicated NK/T cell nasopharyngeal extranodal lymphoma. 

## Case report 

A 60-year-old African American male presented with dysphagia, odynophagia, fatigue, weight loss, and a decline in urine output. He also reported nausea, vomiting, and marked decrease in oral intake. His past history is remarkable for hypertension and NK/T cell nasopharyngeal extranodal lymphoma diagnosed 5 months earlier for which he has received localized radiation therapy, 54 Gy in 2 Gray fractions using intensity modulated radiation therapy (IMRT). Bone marrow biopsy done at diagnosis was negative for any involvement with lymphoma. Chemotherapy had been planned but not started due to missed oncologic appointments. He was not on any prescribed or over-the-counter medications and denied any use of nonsteroidal anti-inflammatory drugs or recent exposure to radiocontrast agents. 

On examination, his initial vital signs were within normal limits. He had dry nasal mucosa with crusting but no bleeding and the nasal septum was intact without perforations. The oral cavity was dry but with no lesions, masses, or ulcers. He had no jaundice, lymphadenopathy, or palpable hepatosplenomegaly. He had dry skin with decreased skin turgor and no extremity edema. 

**Figure 1.**
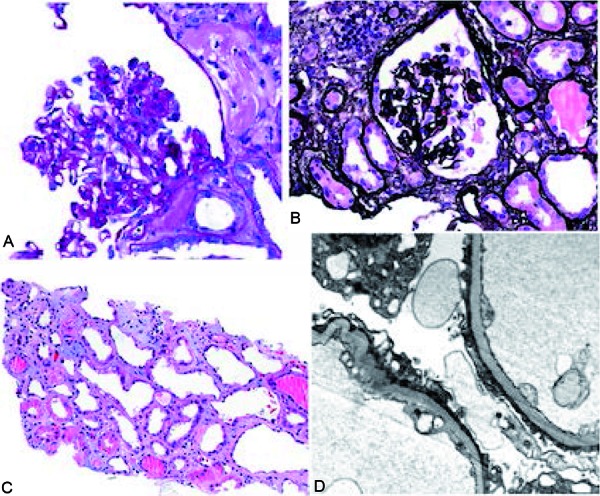
Light microscopy (A – C) and electron microscopy (D) pictures of a renal biopsy specimen. A: PAS stain showing glomerular tuft collapse and overlying epithelial hyperplasia. B: Jones’ silver stain showing glomerular tuft collapse and overlying epithelial hyperplasia. C: Light microscopy showing acute tubular injury. D: Electron microscopy showing diffuse podocyte foot process effacement.

Laboratory data were remarkable for acute kidney injury with a serum creatinine (Cr) of 15.2 mg/dL and blood urea nitrogen (BUN) of 174 mg/dL. He had a baseline Cr of 1.01 mg/dL and BUN 24 mg/dL 3 weeks earlier. Renal ultrasound was unremarkable. Urinalysis of specimen collected from a Foley catheter showed 3+ protein, 3+ blood, occasional granular casts, and 20 – 30 red blood cells (RBCs)/high power field (HPF). A spot urine protein to creatinine ratio was 6.25 gm/gm. His hemoglobin (Hb) was 7.2 g/dL with platelets 132 k/cumm and white blood cell (WBC) count was 2.3 k/cumm with absolute lymphocyte count of 0.3 k/cumm. He became progressively more pancytopenic over the next several days with a nadir WBC count of 1.4 k/cumm, platelet count of 36 k/cumm, and hemoglobin of 6.1 gm/dL. Lactate dehydrogenase (LDH) was found to be elevated at 1,313 U/L and haptoglobin was low (< 8 mg/dL). Coombs’ test was negative. He was started on plasmapheresis for suspected thrombotic microangiopathy pending ADAMSTS13 activity. Peripheral blood smear examination however did not reveal any shistocytes. ADAMTS13 activity was found to be 55% at which time plasmapheresis was stopped. Serology tests for lupus and HIV were negative. Anticardiolipin antibodies, anti-β2-glycoprotein I antibodies and lupus anticoagulant testing were negative. He remained oliguric and was initiated on hemodialysis. During the course of his hospitalization, he started having persistent fever of up to 39 °C. Extensive work-up failed to disclose infectious etiology for his fever. Due to the presence of unexplained renal failure, elevated LDH, and Coombs’ negative hemolytic anemia, the diagnosis of atypical hemolytic uremic syndrome (aHUS) was strongly considered and therapy with complement inhibition was entertained. However this was deferred due to the absence of shistocytes in the peripheral blood, and a kidney biopsy was pursued instead. Kidney biopsy was consistent with collapsing glomerulopathy with evidence of tubular injury, moderate interstitial fibrosis, and tubular atrophy but no pathologic evidence of thrombotic microangiopathy (Figure 1). 

**Figure 2.**
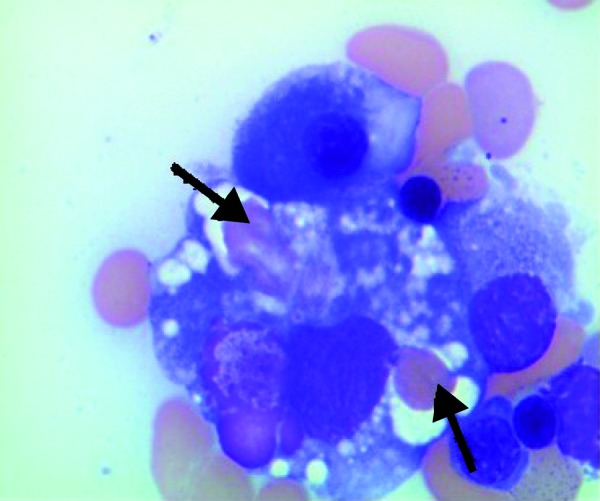
Bone marrow biopsy showing hemophagocytosis. Arrows depict red blood cells engulfed by macrophages in the bone marrow.

The diagnosis of collapsing glomerulopathy in a patient with NK/T cell lymphoma raised suspicion for HPS and a bone marrow (BM) biopsy was obtained. BM biopsy showed increased hemophagocytic activity with many ingested RBCs, neutrophils, and platelets (Figure 2). Additional laboratory data were as follows: fibrinogen: 123 mg/dL, fasting triglyceride level: 361 mg/dL, and ferritin 7,265 ng/mL. The presence of fever, pancytopenia, hypertriglyceridemia, hypofibrinogenemia, markedly elevated ferritin level, and hemophagocytosis on BM biopsy established the diagnosis of HPS in our patient (Table 1). He was started on dexamethasone for HPS, starting with 10 mg/m^2^ daily with plan to taper every 2 weeks. Chemotherapy was not part of his initial regimen for treating HPS due to poor performance status. 

His blood counts started to improve. However he developed cough, dyspnea, and worsening respiratory and mental status. A CT scan of the chest showed new bilateral pulmonary nodules with surrounding ground-glass halo and mild bilateral pleural effusion. Bronchoscopy with bronchoalveolar lavage specimens as well as CT-guided lung biopsy samples came back negative for viral, bacterial, fugal, and mycobacterial pathogens. However the CT-guided lung biopsy findings were consistent with pulmonary involvement with his known NK/T cell lymphoma. Pleural fluid analysis was also positive for malignant cells with a population of CD56-positive T-cells consistent with pleural involvement with his NK/T cell lymphoma. A CT scan of the head was negative for any acute intracranial abnormality and cerebrospinal fluid analysis was negative for infection and malignancy. 

He was subsequently started on PEG-asparaginase, vincristine, and prednisone therapy for his advanced NK/T cell lymphoma. He was also given intrathecal cytarabine. Although there was slight improvement in the size of pulmonary nodules after chemotherapy, he continued to have persistent pleural effusion and worsening overall performance status. As a result, chemotherapy was discontinued and the patient was placed on palliative care. 

## Discussion 

NK/T cell lymphomas are a rare form of Non-Hodgkin’s lymphoma representing ~ 10% of all newly diagnosed peripheral T-cell lymphomas [11]. About 70% present as nasal tumors and the rest as extra-nasal type. Most cases are derived from NK cells with expression of CD56 and cytoplasmic CD3. A large number of patients with nasal type NK/T cell lymphoma present at early stage with single site of disease involvement [12]. The therapy for NK/T cell lymphoma is radiotherapy alone or in combination with chemotherapy, with reported complete response rates of ~ 50 – 60% for early-stage nasal type and a long-term overall survival of ~ 40%. The prognosis of late-stage disease is obviously poor [11, 12]. Association of NK/T cell lymphoma and HPS has been described in the past especially at the relapse setting similar to our patient [13]. However, the combination of collapsing glomerulopathy from HPS complicating NK/T cell lymphoma is very unusual [9]. 

Our patient presented with acute kidney injury (AKI) the etiology of which was initially unclear. Volume depletion due to poor oral intake owing to dysphagia, odynophagia, and vomiting was suspected but was ruled out as he failed to respond to volume expansion. Although there was no nephrotoxic exposure, acute tubular necrosis of ischemic nature resulting from a prolonged prerenal state due to poor intake and vomiting was a definite consideration. However, the severity of his AKI was felt to be out of proportion to the degree of volume depletion. Furthermore, the urine sediment examination was not characteristic of acute tubular necrosis (ATN). The presence of 3+ protein on urinalysis along with a spot urine protein to creatinine ratio of 6.25 gm/gm was also against the diagnosis of ATN. The development of worsening pancytopenia further pointed towards additional pathology. 

The combination of Coombs’ negative hemolytic anemia, thrombocytopenia, elevated LDH, along with AKI made thrombotic microangiopathy (TMA) a strong consideration in our patient [14]. In addition to thrombotic thrombocytopenic purpura (TTP) and typical or atypical hemolytic uremic syndrome, TMA may occur secondary to other disorders such as malignant hypertension, scleroderma, antiphospholipid antibody syndrome, systemic lupus erythematosus, HIV infection, radiation nephropathy, renal allograft rejection, allogeneic HSCT, medications including immunosuppressive and chemotherapeutic agents, infections, and disseminated malignancy [14]. Our patient had a normal blood pressure and had no features of scleroderma. Serologic tests for antiphospholipid antibody syndrome, systemic lupus erythematosus and HIV were also negative. He received only localized radiation and had no history of solid or hematopoietic stem cell transplantation. He had no history of exposure to medications and chemotherapeutic agents known to be associated with TMA. Furthermore a thorough evaluation in our patient failed to disclose any evidence of infection. Although our patient did have malignancy (NK/T cell lymphoma), most described cases of malignancy-associated TMAs were in patients with previously untreated, disseminated mucin-producing adenocarcinomas [15, 16, 17, 18, 19, 20]. Our patient did not have diarrhea and an assay for Shiga toxin-producing *Escherichia coli* was negative. Although he was empirically started on plasma exchange, with no improvement, near normal ADAMTS13 activity level at 55% excludes the diagnosis of classic TTP. aHUS was still a possibility although unlikely in the absence of shistocytes in the peripheral blood [21, 22]. 

Given the uncertainty in the diagnosis of aHUS, particularly the absence of shistocytes in the peripheral blood, a renal biopsy was pursued in our patient. This was thought to be appropriate for pathologic confirmation and exclusion of alternative diagnosis prior to committing the patient to complement inhibition therapy using eculizumab, which is a monoclonal antibody against complement C5 and now widely considered the treatment of choice for aHUS [23]. 

Surprisingly, kidney biopsy was consistent with collapsing glomerulopathy. The diagnosis of collapsing glomerulopathy in our patient with NK/T cell lymphoma raised suspicion for HPS as a possible link between the two [1]. The presence of persistent high grade unexplained fever and pancytopenia, both of which are part of the diagnostic criteria for HPS further led us to pursue this diagnostic possibility [7, 8]. Additional work-up revealed hypofibrinogenemia, hypertriglyceridemia, elevated ferritin level, and increased bone marrow hemophagocytic activity, establishing the diagnosis of HPS based on published guidelines [7, 8]. 

Given the nonspecific nature of its presentation, the diagnosis of HPS can be challenging. The differential diagnosis often includes various infectious, autoimmune, and neoplastic diseases [9]. Interestingly, in our case, a renal biopsy finding of collapsing glomerulopathy in a patient with underlying lymphoma was the first clue to the diagnosis of HPS. 

Data related to renal complications of HPS are limited. Renal involvement has previously been reported in 24 adult cases, mostly as acute renal failure [9]. Nephrotic syndrome has also been described [1, 9, 10]. Collapsing glomerulopathy, minimal change disease, and thrombotic microangiopathy were among the more common findings on renal biopsy [1, 10, 24, 25]. Acute tubulointerstitial nephritis and rapidly progressive glomerulonephritis have also been described [26, 27, 28]. Even though it is the most commonly reported renal biopsy finding, collapsing glomerulopathy complicating HPS is still rare, with only 6 cases reported to date. All were in patients of African descent, as was the case with ours. All but 1 progressed to require dialysis. The etiology for the HPS was leishmaniasis in 1 patient, malaria in another and lymphoma in the remaining 4 patients [1, 9, 10]. 

Renal complications in HPS are believed to result from systemic cytokine burst. Very high amounts of circulating cytokines have been demonstrated in patient with HPS with renal involvement [1]. Primary uncontrolled T-cell activation followed by a cytokine burst involving IFN-γ, TNF-α, IL-6, IL-1β, and other pro-inflammatory cytokines are believed to result in podocyte injuries in genetically predisposed individuals [1]. 

Apart from supportive care, etoposide, dexamethasone, cyclosporine A and, in selected patients, intrathecal therapy with methotrexate are recommended therapeutic options [29]. Subsequent hematopoietic stem cell transplantation (HSCT) is recommended for patients with familial disease or molecular diagnosis, and patients with severe and persistent, or reactivated, disease [1]. Our patient was treated with dexamethasone and although he had improvement in his pancytopenia, he had no improvement in renal function, remained oligoanuric and hemodialysis-dependent. His overall poor functional status precluded a more aggressive approach. 

In summary, HPS is an uncommon syndrome of excessive immune activation clinically presenting with non-specific multi-organ system involvement. Renal complications have rarely been reported. Collapsing glomerulopathy is the most commonly reported finding on renal biopsy, with 6 cases reported in literature so far. Renal prognosis appears to be poor with most patients remaining dialysis-dependent. African decent and lymphoma as an underlying etiology of HPS appear to be common variables among those who went on to develop collapsing glomerulopathy. The diagnosis of collapsing glomerulopathy in a patient with lymphoma should raise suspicion for HPS. Although the outcome was unfavorable, our patient is a great example of complex clinical presentation that requires critical thinking as well as ordering appropriate laboratory and pathology tests until diagnosis is made. 

## Conflict of interest 

The authors have no relevant conflict of interests. 

**Table 1.** Diagnostic criteria for hemophagocytic syndrome used in the HLH-2004 trial*.

The diagnosis of hemophagocytic syndrome may be established when:
A. Molecular diagnosis consistent with HLH: pathologic mutations of *PRF1*, *UNC13D*, *Munc18-2*, *Rab27a*, *STX11*, *SH2D1A*, or *BIRC4*
Or
B. Five of the 8 criteria listed below are fulfilled:
1. Fever ≥ 38.5 °C
2. Splenomegaly
3. Cytopenias (affecting at least 2 of 3 lineages in the peripheral blood): Hemoglobin < 9 g/dL Platelets < 100 × 10^3^/mL Neutrophils < 1 × 10^3^/mL
4. Hypertriglyceridemia (fasting > 265 mg/dL) and/or hypofibrinogenemia (< 150 mg/dL)
5. Hemophagocytosis in bone marrow, spleen, lymph nodes, or liver
6. Low or absent NK-cell activity
7. Ferritin > 500 ng/mL
8. Elevated sCD25 (α-chain of sIL-2 receptor)

*Adopted from reference [8].

## References

[1] Thaunat O Delahousse M Fakhouri F Martinez F Stephan JL Noël LH Karras A Nephrotic syndrome associated with hemophagocytic syndrome. Kidney Int. 2006; 69: 1892–1898. 16557222 10.1038/sj.ki.5000352

[2] Fujiwara F Hibi S Imashuku S Hypercytokinemia in hemophagocytic syndrome. Am J Pediatr Hematol Oncol. 1993; 15: 92–98. 8447564 10.1097/00043426-199302000-00012

[3] Larroche C Mouthon L Pathogenesis of hemophagocytic syndrome (HPS). Autoimmun Rev. 2004; 3: 69–75. 15003190 10.1016/S1568-9972(03)00091-0

[4] Filipovich A McClain K Grom A. Histiocytic disorders: recent insights into pathophysiology and practical guidelines. Biol Blood Marrow Transplant. 2010; 16: S82–S89. 19932759 10.1016/j.bbmt.2009.11.014

[5] Imashuku S Differential diagnosis of hemophagocytic syndrome: underlying disorders and selection of the most effective treatment. Int J Hematol. 1997; 66: 135–151. 9277044 10.1016/s0925-5710(97)00584-7

[6] Reiner AP Spivak JL Hematophagic histiocytosis. A report of 23 new patients and a review of the literature. Medicine (Baltimore). 1988; 67: 369–388. 3054418

[7] Henter JI Elinder G Ost A Diagnostic guidelines for hemophagocytic lymphohistiocytosis. Semin Oncol. 1991; 18: 29–33. 1992521

[8] Henter JI Horne A Aricó M Egeler RM Filipovich AH Imashuku S Ladisch S McClain K Webb D Winiarski J Janka G HLH-2004: Diagnostic and therapeutic guidelines for hemophagocytic lymphohistiocytosis. Pediatr Blood Cancer. 2007; 48: 124–131. 16937360 10.1002/pbc.21039

[9] Ramos-Casals M Brito-Zerón P López-Guillermo A Khamashta MA Bosch X Adult haemophagocytic syndrome. Lancet. 2014; 383: 1503–1516. 24290661 10.1016/S0140-6736(13)61048-X

[10] Niang A Niang SE Ka HF Ka MM Diouf B Collapsing glomerulopathy and haemophagocytic syndrome related to malaria: a case report. Nephrol Dial Transplant. 2008; 23: 3359–3361. 18676345 10.1093/ndt/gfn427

[11] Au WY Weisenburger DD Intragumtornchai T Nakamura S Kim WS Sng I Vose J Armitage JO Liang R Clinical differences between nasal and extranasal natural killer/T-cell lymphoma: a study of 136 cases from the International Peripheral T-Cell Lymphoma Project. Blood. 2009; 113: 3931–3937. 19029440 10.1182/blood-2008-10-185256

[12] Vose J Armitage J Weisenburger D International peripheral T-cell and natural killer/T-cell lymphoma study: pathology findings and clinical outcomes. J Clin Oncol. 2008; 26: 4124–4130. 18626005 10.1200/JCO.2008.16.4558

[13] Takahashi N Miura I Chubachi A Miura AB Nakamura S A clinicopathological study of 20 patients with T/natural killer (NK)-cell lymphoma-associated hemophagocytic syndrome with special reference to nasal and nasal-type NK/T-cell lymphoma. Int J Hematol. 2001; 74: 303–308. 11721967 10.1007/BF02982065

[14] George JN How I treat patients with thrombotic thrombocytopenic purpura: 2010. Blood. 2010; 116: 4060–4069. 20686117 10.1182/blood-2010-07-271445

[15] Hostetter AL Tubbs RR Ziegler T Gephardt GN McMahon JT Schreiber MJ Chronic glomerular microangiopathy complicating metastatic carcinoma. Hum Pathol. 1987; 18: 342–348. 3104197 10.1016/s0046-8177(87)80163-6

[16] Chang JC Naqvi T Thrombotic thrombocytopenic purpura associated with bone marrow metastasis and secondary myelofibrosis in cancer. Oncologist. 2003; 8: 375–380. 12897334 10.1634/theoncologist.8-4-375

[17] Francis KK Kalyanam N Terrell DR Vesely SK George JN Disseminated malignancy misdiagnosed as thrombotic thrombocytopenic purpura: A report of 10 patients and a systematic review of published cases. Oncologist. 2007; 12: 11–19. 10.1634/theoncologist.12-1-11 17227897

[18] Werner TL Agarwal N Carney HM Rodgers GM Management of cancer-associated thrombotic microangiopathy: what is the right approach? Am J Hematol. 2007; 82: 295–298. 17034030 10.1002/ajh.20783

[19] George JN Systemic malignancies as a cause of unexpected microangiopathic hemolytic anemia and thrombocytopenia. Oncology (Williston Park). 2011; 25: 908–914. 22010388

[20] Robier C Neubauer M Beham-Schmid C Sill H Thrombotic microangiopathy and disseminated intravascular coagulation associated with carcinocythemia in a patient with breast cancer. J Clin Oncol. 2011; 29: e825–e826. 22025152 10.1200/JCO.2011.36.7433

[21] Burns ER Lou Y Pathak A Morphologic diagnosis of thrombotic thrombocytopenic purpura. Am J Hematol. 2004; 75: 18–21. 14695628 10.1002/ajh.10450

[22] Laurence J. Atypical hemolytic uremic syndrome (aHUS): making the diagnosis. Clin Adv Hematol Oncol. 2012; 10: 1–12. 23187605

[23] Kaplan BS Ruebner RL Spinale JM Copelovitch L Current treatment of atypical hemolytic uremic syndrome. Intractable Rare Dis Res 2014; 3: 34–45. 10.5582/irdr.2014.01001 PMC4204535 25343125

[24] Chiang WC Wu MS Tsai CC Lin SL Tsai TJ Hsieh BS Thrombotic microangiopathy in hemophagocytic syndrome: a case report. J Formos Med Assoc. 2002; 101: 362–367. 12101856

[25] Ardalan MR Shoja MM Tubbs RS Esmaili H Keyvani H Postrenal transplant hemophagocytic lymphohistiocytosis and thrombotic microangiopathy associated with parvovirus b19 infection. Am J Transplantat. 2008; 8: 1340–1344. 10.1111/j.1600-6143.2008.02244.x 18522549

[26] Cho E Cha I Yoon K Yang HN Kim HW Kim MG Jo SK Cho WY Kim HK Hemophagocytic syndrome in a patient with acute tubulointerstitial nephritis secondary to hepatitis A virus infection. J Korean Med Sci. 2010; 25: 1529–1531. 20890439 10.3346/jkms.2010.25.10.1529 PMC2946668

[27] Hoshino C Satoh N Sugawara S Kuriyama C Kikuchi A Ohta M Community-acquired Staphylococcus aureus pneumonia accompanied by rapidly progressive glomerulonephritis and hemophagocytic syndrome. Intern Med. 2007; 46: 1047–1053. 17603250 10.2169/internalmedicine.46.6378

[28] To KF Chan PK Chan KF Lee WK Lam WY Wong KF Tang NL Tsang DN Sung RY Buckley TA Tam JS Cheng AF Pathology of fatal human infection associated with avian influenza A H5N1 virus. J Med Virol. 2001; 63: 242–246. 11170064 10.1002/1096-9071(200103)63:3<242::aid-jmv1007>3.0.co;2-n

[29] Jordan MB Allen CE Weitzman S Filipovich AH McClain KL How I treat hemophagocytic lymphohistiocytosis. Blood. 2011; 118: 4041–4052. 21828139 10.1182/blood-2011-03-278127 PMC3204727

